# Hydrochemical characteristics and irrigation suitability of surface water in the Syr Darya River, Kazakhstan

**DOI:** 10.1007/s10661-019-7713-8

**Published:** 2019-08-16

**Authors:** Weiyan Zhang, Long Ma, Jilili Abuduwaili, Yongxiao Ge, Gulnura Issanova, Galymzhan Saparov

**Affiliations:** 1 State Key Laboratory of Desert and Oasis Ecology, Xinjiang Institute of Ecology and Geography, Chinese Academy of Sciences, Urumqi, 830011 China; 2 Research Center for Ecology and Environment of Central Asia, Chinese Academy of Sciences, Urumqi, 830011 China; 3 University of Chinese Academy of Sciences, Beijing, 100049 China; 4 Faculty of Geography and Environmental Sciences, Al-Farabi Kazakh National University, Ave. Al-Farabi, 75 V, Almaty, Kazakhstan 050040; 5 Uspanov Kazakh Research Institute of Soil Science and Agricultural Chemistry, Almaty, Kazakhstan 050060

**Keywords:** Hydrochemical characteristics, Irrigation suitability, Surface water, Syr Darya, Kazakhstan, Statistic analysis

## Abstract

The surface water hydrochemistry of the Syr Darya River in Kazakhstan was investigated at 39 locations to analyze regional hydrochemical characteristics and evaluate the irrigation suitability of the studied regions. The cations in the surface water are mainly Na^+^, Ca^2+^, and Mg^2+^, while the anions are mainly SO_4_^2−^. The main hydrochemical type is Ca-Mg-SO_4_-Cl. From the perspective of natural factors, the hydrochemical characteristics in the study area are derived from the dual effects of rock weathering and evaporation-concentration; however, the influence of anthropogenic factors include industrial and agricultural production near the river and the inflow of urban domestic sewage on the hydrochemical characteristic is also present. The irrigation suitability evaluation of the surface water based on SAR, Na% and KI showed that the majority of the water is suitable for irrigation, and from the TDS content analysis, 71.43% of the samples are in a critical state, indicating that the influence of TDS concentration on irrigation suitability cannot be ignored. The results have practical significance for maintaining the sustainable use of water resources in the Syr Darya River.

**Electronic supplementary material:**

The online version of this article (10.1007/s10661-019-7713-8) contains supplementary material, which is available to authorized users.

## Introduction

The hydrochemical characteristics of water are indicative of the climate and environment in the area where the river flows. As an important factor determining the use of water for domestic, irrigation, or industrial purposes, the hydrochemical characteristics are of great significance to the sustainable management of water resource utilization and the protection and construction of the ecological environment. Chemical ions in water are regarded as natural “tracers”, and the analysis of the main ion composition in water can be used to identify and control the basic processes affecting the chemical composition of the water, such as the weathering of rocks in river basins, the evaporation and concentration of water bodies, and the input of atmospheric compounds via precipitation (Han and Liu 2004; Sadashivaiah and Ranganna 2008; Dinka et al. 2015). Meanwhile, anthropogenic activities such as irrigation have a great influence on the water salinity, many salts, and agrochemical loadings will affect the water quality with irrigation return flows (Isidoro and Aragüés 2007). Ions and metals are introduced from fertilizers and other agrochemicals (Laar et al. 2011), in this study, it analyzes the suitability of water irrigation suitability from its hydrochemical characteristics (Kirda 1997; Nishanthiny et al. 2010; Mohammed Muthanna 2011).

The Syr Darya is the longest river in Central Asia, and the middle and lower reaches of the river are located in Kazakhstan. Under the dual burden of a water shortage caused by the uneven use of water at upstream and downstream locations and pollutant accumulation in the upper reaches, river water is still needed to maintain the productivity and daily life of the residents and the ecological balance along the river; as a result, the middle and lower reaches of the river are become the most sensitive area with regard to the ecology and environment of the whole basin. As early as the 1980s and 1990s, due to the lack of large-scale renovations and regular defensive measures in the Syr Darya River, many water distribution facilities and channels were destroyed by the spring ice drift and the backwaters of the lake system. Overgrowth, sedimentation, and the collapse of riverside terraces reduced the capacity of the river, and temporary dams on the channels were often washed away, causing the water to flow back to the Syr Darya River, negatively affecting the salinity conditions of the water system. In addition, the development of irrigation has shifted the ion content in the water of the Syr Darya River from calcium carbonate to sodium-magnesium and sulfate-chloride (Kipshakbaev et al. 2010). A total of 20 million tons of salt per year enters the Syr Darya River by drained return flow, which increase the salt content of the river from 300 to 600 mg L^−1^ upstream to 3000 mg L^−1^ downstream of the Fergana Valley, MgSO_4_, Ca (HCO_3_)_2_, NaCl, and CaSO_4_ are commonly found in the salt composition (SIC ICWC et al. 2011). Based on the results of many years of research in recent decades regarding the water of the lower reaches of Syr Darya, the mineralization degree of the surface water is as high as 900–1100 mg L^−1^, and there is an increased concentration of sulfate compounds, up to 40–45% (Bekbaev and Kazykenova 2003; Kenjebayeva 2015). In addition, 100% of the irrigated area in Kyzylorda and 67.8% of the area in South Kazakhstan is moderately or highly saline, which results in large-scale crop reductions (Murray-Rust et al. 2017).

Therefore, it is urgent and important to study the hydrochemical characteristics and irrigation suitability of the Syr Darya River in Kazakhstan. This study considers the main ion content of the surface water of the Syr Darya River in Kazakhstan, analyzes the water characteristics and the relationship between each component and each ion species, and uses the sodium absorption ratio, Na%, and Kelly’s index (KI) to evaluate the suitability of irrigation. The results have a substantial influence on understanding the current situation of regional surface water hydrochemistry and the influence of these parameters on agriculture and provide data support for regional salinization treatment.

## Materials and methods

### Description of the study area

The Aral-Syr Darya basin in Kazakhstan covers an area of 345000 km^2^ and comprises two states: South Kazakhstan and Kyzylorda (UNDP 2004; Nugumanova et al. 2017). The length of the Syr Darya River from the Shardara reservoir to the Aral Sea in Kazakhstan is 1627 km; the largest tributary of the Syr Darya is the Arys River. A total of 38.1% of the water in Kazakhstan is accounted for by the runoff of the Syr Darya River, which has an average annual flow of 179 × 10^8^ m^3^, of which 137 × 10^8^ m^3^ is derived from other countries. The volume consumed is 69 × 10^8^ m^3^, and the available water resource volume is 110 × 10^8^ m^3^ (Deng 2012; Micklin 2014). Kazakhstan, located inland, is weakly affected by the ocean and has a typical arid continental climate. It is hot and dry in summer and cold in winter. The average temperature in January is − 19 to approximately − 4 °C, the average temperature in July is 19~26 °C, and the absolute maximum and minimum temperatures are 45 °C and − 45 °C, respectively. The warm period in South Kazakhstan, with an average daily air temperature above 0 °С, lasts up to 10 months; the hottest month in this period is July, and the average daily temperature in this month reaches 29 °С (Magay 2013). The annual precipitation in most parts of the country is less than 250 mm. Precipitation in desert areas is less than 100 mm. There is less vegetation in arid areas, and land cannot be cultivated without artificial irrigation. The climate in the eastern and southeastern foothills is humid, with a precipitation of 400–600 mm; precipitation in the mountainous area can reach up to 90 mm, and the annual precipitation in the southern plains of northern Siberia is 300 mm or greater. The land can be cultivated without irrigation. The main agricultural areas of Kazakhstan are concentrated in the northern plains and the eastern and southeastern foothills; this distribution is closely associated with the climate (Hong 2015).

The Aral-Syr Darya basin is one of the eight main basins for water economy in Kazakhstan and ranks third in area. The regional population accounts for 16% of the total population of Kazakhstan, within this region, the urban population accounts for 46.43%, and the rural population accounts for 53.57% (Issanova et al. 2018). Irrigation as the biggest water consumer in the region, which consumed 68.03% of the regional total water withdrawal in 2011, and the total value of agricultural products accounts for 10.09% of regional GDP (Dynamics of General Indicators of the Aral Sea Basin states 2015). The main geomorphological characteristics of the study area are the Turan Lowland and an orogenic belt, and the tectonic geomorphology type is an intermediate-scale fault-bounded basin an intermediate block activation depression basin.

### Sample collection and preparation

**Fig. 1:**
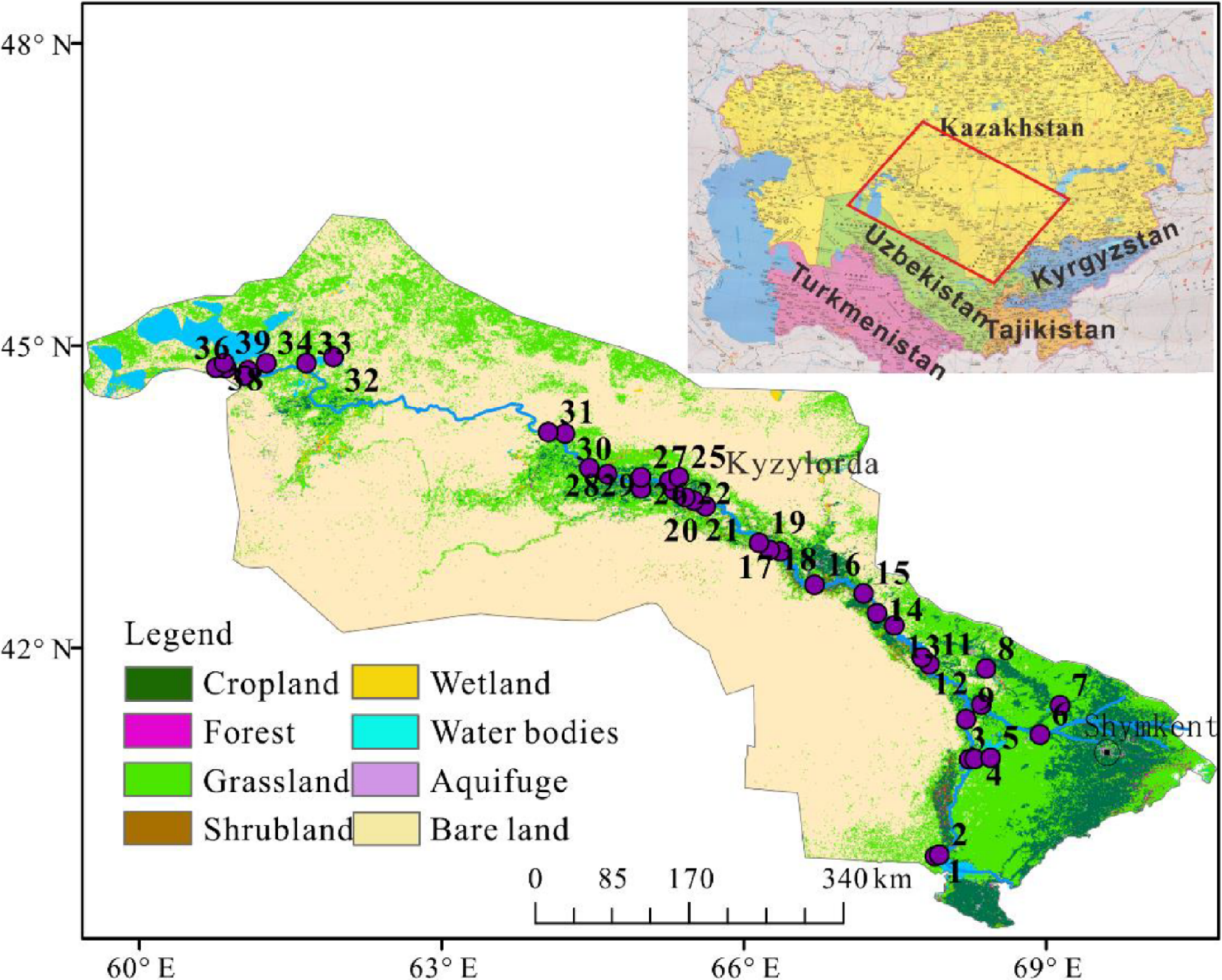
Geographical locations of study area and samples

This study focused on the Syr Darya River within Kazakhstan. Thirty-nine surface water samples were collected along the Syr Darya, starting from the Shardara reservoir to the North Aral Sea entrance, in June 2017 (Fig. 1). The sampling points were located on bridges or other floating structures suitable for the collection of water samples. Approximately 250 mL of water was collected from each point and placed in a clean polyethylene container. All the samples were stored in a 4 °C refrigerator until analysis (Table 1).

**Table 1:** The description of sample point location

Sample number	Sample position	Sample number	Sample position
1	Shardara reservoir	21	Main channel through the residential area
2	Main channel	22	Small tributary beyond farmland
3	Main channel	23	Small tributary beyond farmland
4	Small tributary	24	Main channel
5	A catchment for water from sample No.4	25	A lake
6	Arys river	26	Small tributary
7	Bugunskoye water reservoir	27	Small tributary
8	Shoshkakol lake	28	Small tributary
9	Main channel	29	Main channel
10	A bifurcated channel through the residential area of Shaulder	30	Small tributary
11	Main channel	31	Main channel
12	Main channel	32	Qamystybas lake
13	Main channel	33	Qamystybas lake
14	Koylekata lake, receiving irrigation water	34	Small lake
15	Main channel	35	Small lake
16	Main channel	36	Main channel
17	Main channel	37	Main channel
18	Small tributary	38	Kokaral dam
19	Small tributary	39	North Aral Sea
20	Main channel		

### Analytical methods for chemical parameters

The major ions in water samples were treated and analyzed at the Sino-Kazakh joint test center in Almaty, Kazakhstan. The dissolved water samples were filtered (through a ca. 0.45-μm membrane) for analysis for Na^+^ and K^+^ (by flame spectrometry), Ca^2+^ and Mg^2+^ (by EDTA titration), HCO_3_^−^ and CO_3_^2−^ (by acid titration), Cl^−^ (by AgNO_3_ titration), and SO_4_^2−^ (by BaCl_2_ titration), Total dissolved solid (TDS, by gravimetry). The basic physical and chemical properties of the water samples, including pH and dissolved oxygen (DO) were measured by a portable multi-parameter water quality analyser (HQ40d, Hach Corporation, USA) at the same time. The analytical precision of the measurement of ions was determined by calculating the ion balance error, which was within 5%.

### Assessment method

#### Irrigation suitability assessment

Irrigation waters contain a large number of chemical constituents. The main characteristics for evaluating water quality and determining whether water can be used for irrigation are the following: the relative ratio of sodium to other cations, known as the sodium percentage (Na%), sodium absorption ratio (SAR), Kelly index (KI) (Kelley 1940; Shaki and Adeloye 2006; Nishanthiny et al. 2010; Ravikumar and Somashekar 2012; Zhang et al. 2012; Ghazaryan and Chen 2016).

Water containing high concentrations of Na^+^ poses greater hazards when used for irrigation because Na^+^ is absorbed into the soil, causing soil polymer dispersion and leading to a decrease in permeability. The formula is as follows:

1 $$ \mathrm{Na}\%=\frac{{\mathrm{Na}}^{+}}{{\mathrm{Ca}}^{2+}+{\mathrm{Mg}}^{2+}+{\mathrm{Na}}^{+}+{\mathrm{K}}^{+}}\times 100\% $$

SAR represents the relative activity of Na^+^ in soil exchange reactions and is used to evaluate the degree of alkalization of irrigation water (Ayers and Westcot 1985). Irrigation with high-SAR water can cause soil slabs. The formula is as follows:

2 $$ \mathrm{SAR}=\frac{{\mathrm{Na}}^{+}}{\sqrt{\left({\mathrm{Ca}}^{2+}+{\mathrm{Mg}}^{2+}\right)/2}} $$

The sodium content in irrigation water affects the exchange of Ca^2+^ and Mg^2+^ in soil clay granules, resulting in reduced permeability of the soil, internal drainage, and air circulation. KI is also used to classify the grade of irrigation water, and when the value is less than 1, the water is suitable for irrigation. The formula is as follows:

3 $$ \mathrm{KI}=\frac{{\mathrm{Na}}^{+}}{{\mathrm{Ca}}^{2+}+{\mathrm{Mg}}^{2+}} $$

where all the ionic concentrations are expressed in milliequivalents per liter (meq L^−1^) of the respective ions.

## Results and discussion

### Hydrochemical characteristics

The hydrochemical characteristic of surface water in the Syr Darya River was analyzed, and the results are shown in Table 2. The pH values of the study area ranged from 7.95~9.31, with a mean value of 8.32, which is weakly alkaline. The dissolved oxygen (DO) values ranged from 7.98~14.45, with a mean value of 8.96. The total dissolved solids (TDS) values indicated that the salinity of surface water in the study area varied between 342.00 mg L^−1^ and 4014.00 mg L^−1^, with a mean value of 1144.49 mg L^−1^, 94.87% of samples were above the limit of 500 mg L^−1^ which indicating the presence of slightly salinity and related to the problem such as hardness (Herojeet et al. 2013), and approximately 25.64% of samples were considered as brackish water (TDS > 1000 mg L^−1^).

**Table 2:** Descriptive statistics of Syr Darya River hydrochemical parameters

Item	pH	DO	TDS	HCO_3_^-^	CO_3_^2-^	Cl^-^	SO_4_^2-^	Ca^2+^	Mg^2+^	Na^+^	K^+^
Mean	8.32	8.96	1144.49	169.31	4.51	142.95	491.01	105.24	75.69	127.68	7.20
Max	9.31	14.45	4014.00	244.00	14.00	1053.00	2120.00	300.00	426.00	586.00	38.00
Min	7.95	7.98	342.00	83.00	0.00	16.00	30.00	40.00	17.00	23.00	1.00
SD	0.25	1.28	908.76	30.09	3.92	216.18	466.22	53.14	90.95	137.53	7.85
CV (%)	2.95	14.32	79.40	17.77	86.85	151.23	94.95	50.49	120.16	107.72	108.95

pH is dimensionless, and the units of the remaining parameters are mg·L^−1^

From the average ion concentrations, the main anions were dominated by SO_4_^2−^, followed by HCO_3_^−^ and Cl^−^, and the least abundant anion was CO_3_^2−^, the average content of SO_4_^2−^ in the study area was higher than the limit value of fishery 100 mg L^−1^ but lower than the standard value of drinking water 500 mg L^−1^, it will affect the safe of fishery; the main cations were dominated by Na^+^, Ca^2+^ and Mg^2+^, and the content of K^+^ was relatively small. The coefficient of variation (CV) is the percent standard deviation relative to the average value of each element and reflects the degree of dispersion of elements among the samples. The results showed that the CVs of all ions were greater than 10%; in particular, the CVs of Cl^−^, Mg^2+^, Na^+^, and K^+^ were over 100%, which indicated strong spatial variability, showing that these ions are sensitive factors that change with the environment and determine the main variables of salinization.

**Fig. 2:**
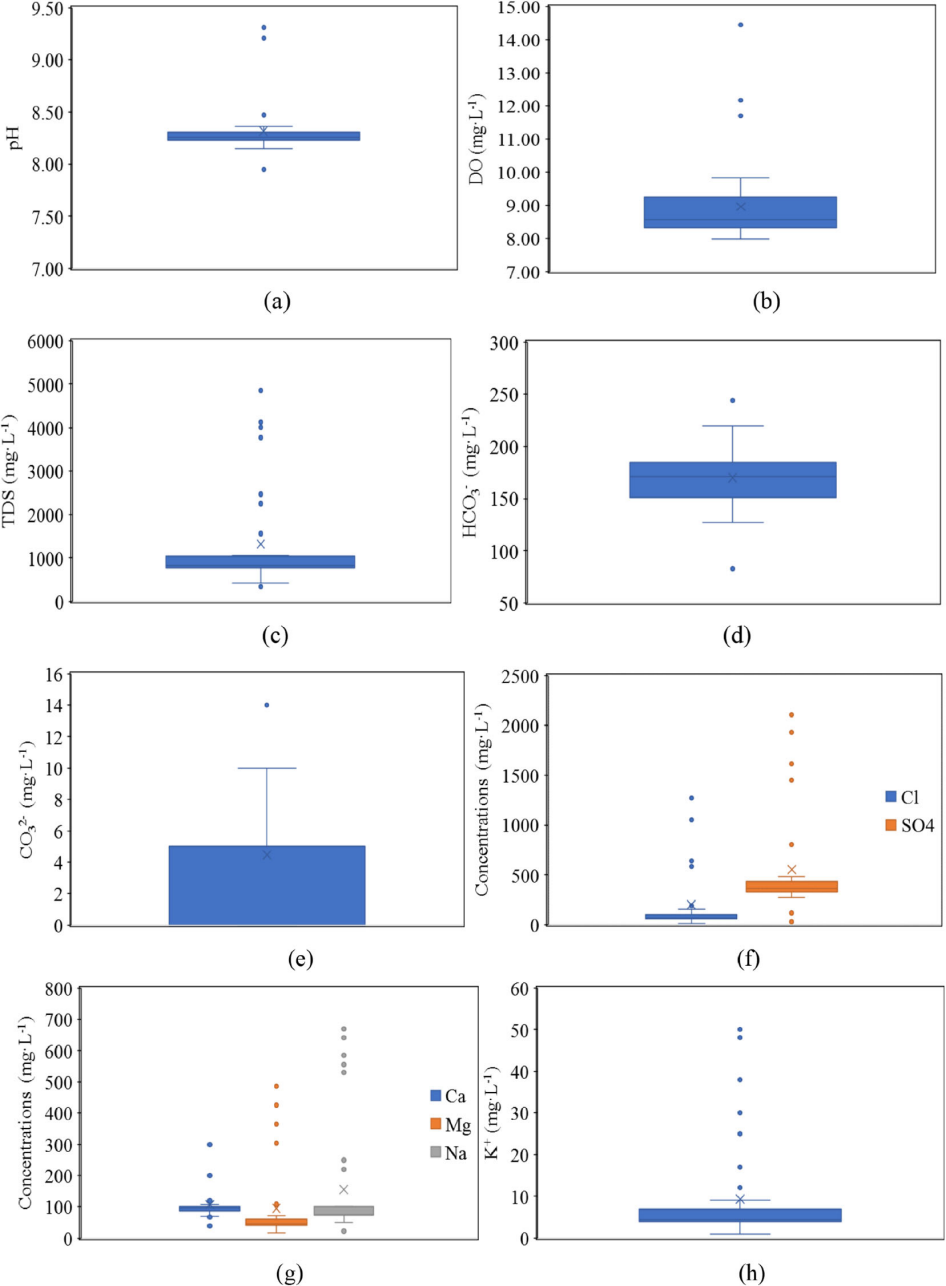
Box diagram of hydrochemical characteristics

From the results of descriptive statistics of Syr Darya river hydrochemical parameters, it is found that the extreme value difference of all parameters are relatively large, so the outlier value of each parameter is analyzed by box plot (Fig. 2), Box plot analysis is based on actual data, showing the original distribution of data in a real and intuitive way, it is based on the quartile and the interquartile range to determine the outliers, the results are more objective and have certain advantages in identifying outliers( Wang 2011). As shown in Fig. 2a and b, there are 4 outliers of the pH and 3 outliers of the DO, the normal value ranged from 7.95 to 8.47 and 7.98 to 9.83 mg L^−1^, respectively, the difference in pH and DO content between samples is little. As shown in Fig. 2c–e, there are 9 outliers for TDS, the normal value ranged from 425.00 to 1059.00 mg L^−1^, which almost belong to the fresh water (< 1000 mg L^−1^)2 outliers for HCO_3_^-^, and the normal value ranged from 127.00 to 220.00 mg·L^−1^, and 1 outlier for CO_3_^2−^, the normal value ranged from 0.00 to 10.00 mg L^−1^. As shown in Fig. 2f, there are 7 outliers for Cl^-^, the normal value ranged from 16.00 to 159.00 mg L^−1^, and 10 outliers for SO_4_^2-^, the normal value ranged from 277.00 to 479.00 mg L^−1^, which are all higher than the standard (100 mg L^−1^) recommended for fishing (the list of fishery standards 1999). As shown in Fig. 2g, there are 10 outliers for Ca^2+^, 8 outliers for Mg^2+^ and 10 outliers for Na^+^, and the normal value ranged from 70 to 108 mg L^−1^, 17 to 73 mg L^−1^, and 50 to 100 mg L^−1^, respectively. As shown in Fig. 2h, there are 7 outliers for K^+^, the normal value ranged from 1.00 to 9.00 mg L^−1^.

The determination of the handling mode of outliers requires further analysis about the causes of outliers, because of the highly variable with space of the water quality monitoring data, that is, they are closely related to the surrounding environmental characteristics of the location of outliers, and the occurrence of outliers often implies some changes in the pollution situation (Deng 1995). Therefore, it is necessary to analyze the causes of outliers in combination with the location.

**Fig. 3:**
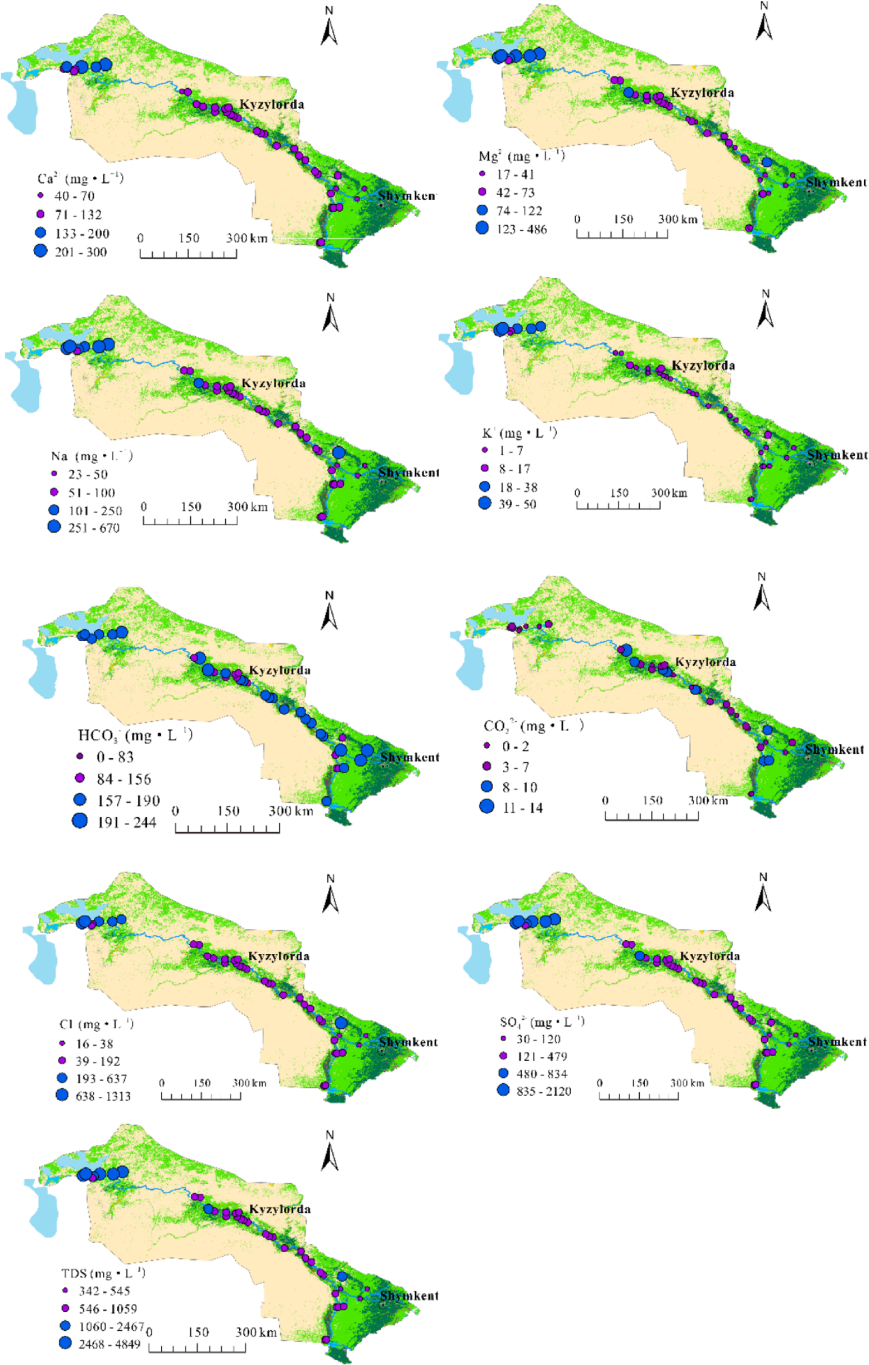
Spatial distribution of hydrochemical characteristics of surface water in the Syr Darya River, Kazakhstan

The spatial distribution characteristics of the chemical components of water in the Syr Darya are shown in Fig. 3. Medium–high values of all ions except HCO_3_^−^ and CO_3_^2−^ are mainly concentrated in the area close to the North Aral Sea. High concentrations of HCO_3_^−^ and CO_3_^2−^ are evenly distributed in the watershed, which is consistent with the distribution of regional farmland; the addition of these two ions to soil has a large impact on crop productivity (Karlykhanov and Toktaganova 2016). The spatial distribution characteristics of TDS are obvious: high values are concentrated in the North Aral Sea region due to the strong evaporation of water and wind erosion that the salt accumulation effect in this area is stronger. In addition, there is an abnormal point (sample 8) near the Arys River with respect to the spatial distribution characteristics of Mg^2+^, Na^+^, Cl^−^, CO_3_^2−^, and TDS, this point is located in the Bugunskoye water reservoir, which was built for irrigation according to the Arys–Turkestan scheme, the reservoir accumulates the runoff from the Bugun River and part of the runoff from the Arys River, which is delivered by the Arys canal and has a high salt content (Starodubtsev 2012).

**Fig. 4:**
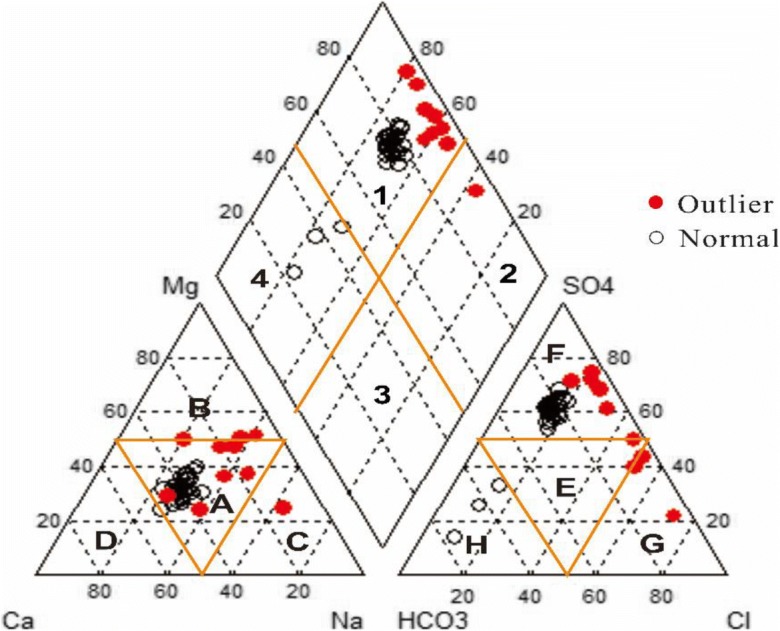
Piper diagram of the chemical facies of surface water in the study area (1, CaMgSO_4_Cl; 2, NaSO_4_Cl; 3, NaHCO_3_; 4, CaMgHCO_3_; A, mixed zone; B, Mg; C, NaK; D, Ca; E, mixed zone; F,SO_4_; G, Cl; H, HCO_3_.)

The geochemical classification of water mainly depends on the concentrations of various major ions and their correlations. The Piper diagram (Piper 1944) is a commonly used and very effective method for classifying water chemical types based on the basic geochemical characteristics of major ions. The chemical data for the surface water samples collected from the study area are plotted in a Piper diagram (Fig. 4). According to this figure, there were three main hydrochemical types of surface water in the study area, including the Ca-Mg-SO_4-_Cl type, which accounted for 89.74% of water samples; the Ca-Mg-HCO_3_ type, which accounted for 7.69% (3 samples) of water samples; and the Na-SO_4_-Cl type, which accounted for only 2.56% of water samples. Most of the surface water in the study area was characterized by significantly more alkaline earth elements (Ca^2+^+Mg^2+^) than alkali elements (Na^+^+K^+^), and strong acids (SO_4_^2−^ + Cl^−^) exceeded weak acids (HCO_3_^−^).

In the lower left corner of the cation triangle, there were three types of cations; most of the samples were located in the mixed zone, which accounted for 92.31%, Mg^2+^ accounted for 5.13% and Na^+^+K^+^ accounted for 2.56%. In the lower right corner of the anion triangle, there were three types of anions, the main type of anion was SO_4_^2−^, which accounted for 84.62%, followed by Cl^−^ (7.69%), and HCO_3_^−^ (7.69%).

The outlier group samples are located in the area where the cumulative effect is strong. According to the piper diagram analysis, the main geochemical classification of outlier group was Ca-Mg-SO_4_-Cl, and the main anion was SO_4_^2−^, and the main cation was located in mixed zone.

There are many irrigation areas along the Syr Darya in Kazakhstan, most of which are located in the Kyzylorda region. According to the results of a hydrogeological survey in the Kyzylorda region, the soil texture in the region is sandy clay (Mustafayev et al. 2014; Chen et al. 2017), which has good water permeability, poor water retention, and poor fertilizer retention performance. Irrigation water diversion projects and groundwater extraction, among other reasons, have caused the surface water and groundwater to have a high reuse rate and strong mixing effect. Irrigation drainage water with salt and fertilizers is discharged into the river; in addition, leachate filtration and transpiration concentration in runoff, sulfur-containing industrial wastewater and domestic sewage, result in a slightly salty water belt dominated by sulfate (Li et al. 2010).

**Fig. 5:**
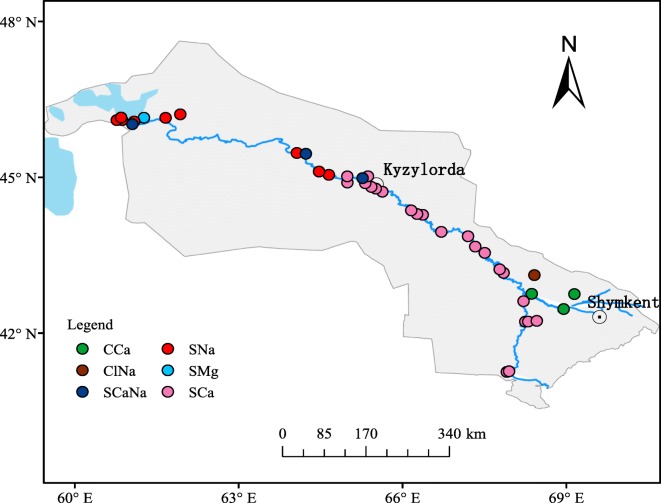
Spatial distribution of hydrochemical classifications

The Piper diagram depicts the overall main hydrochemical types of surface water in the study area, and Arliekin’s classification method was used to calculate the Piper diagram data and indicate the hydrochemical type of each sample. The Arliekin method produces classifications according to the dominant components of water and the ratios between ions. Consequently, all samples were subdivided into six types: the main hydrochemical type was sulfide calcium water (S^Ca^), accounting for 56.41%, followed by S^Na^ (23.08%), C^Ca^ (7.69%), S^CaNa^ (7.69%), S^Mg^ (2.56%), and Cl^Na^ (2.56%). About 89.74% (35 samples) of water samples were type II, and about 10.26% (samples 8, 32, 38, and 39) of water samples were type III, which is high-salinity water. The spatial distribution of the hydrochemical classifications is shown in Fig. 5. The main hydrochemical type of SO_4_^2−^–Ca^2+^ was distributed from Shardara reservoir to Kyzylorda city; SO_4_^2−^–Na^+^, SO_4_^2−^–Mg^2+^ and SO_4_^2−^–Ca^2+^–Na^+^ were mainly concentrated in the area from Kyzylorda city to North Aral Sea, and the types of HCO_3_^−^–Ca^2+^ and Cl^−^–Na^+^ were distributed along the tributary Arys River. The Arys River is located in the foothill plain and valley area, and the soil geography of this region belongs to a bicarbonate-based freshwater belt (Chen et al. 2017), which might be a reason why the identified ions are concentrated in this region.

**Fig. 6:**
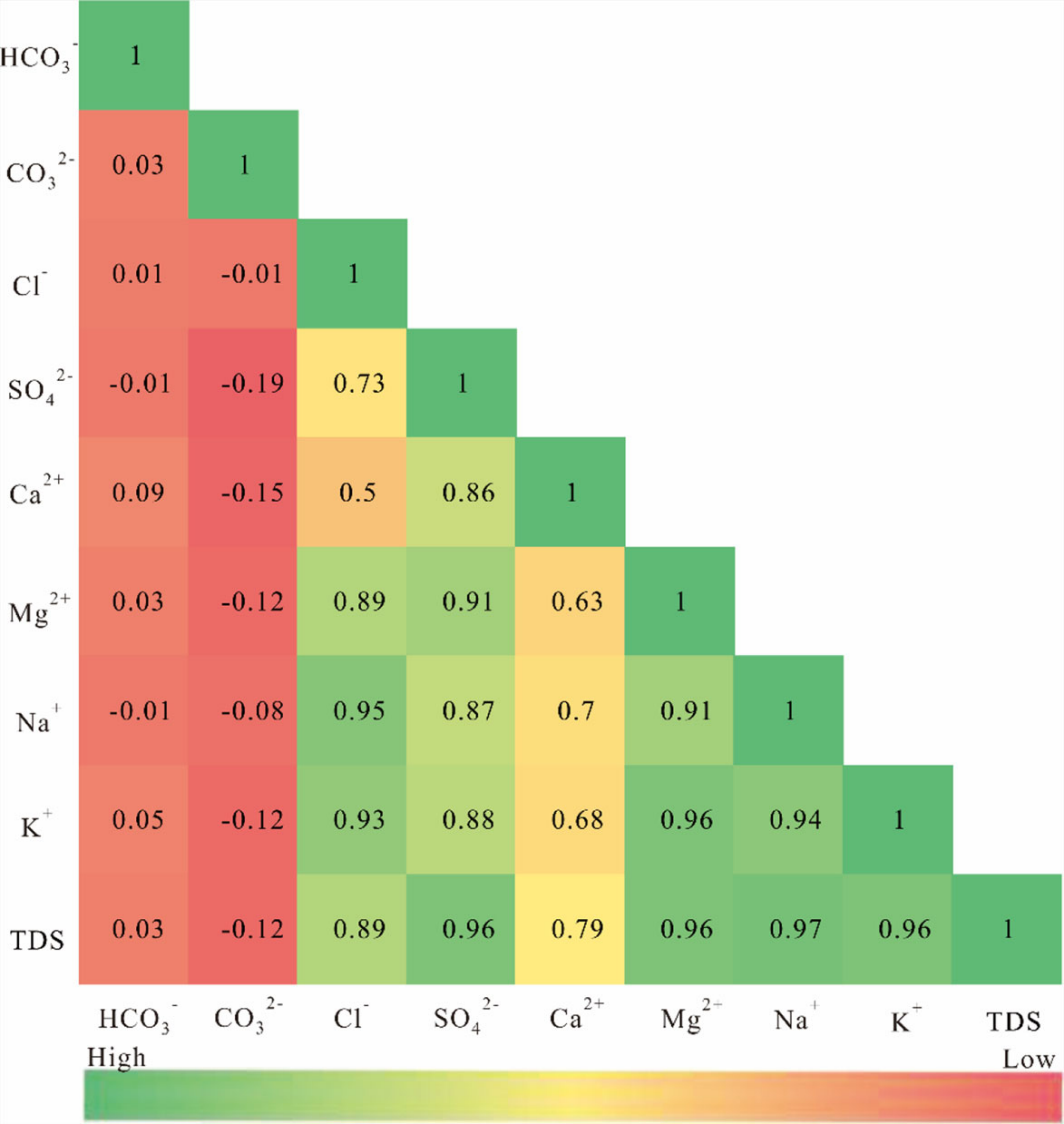
Correlation matrix of hydrochemical parameters

Correlation analysis is a statistical method used to measure the degree of correlation between two or more correlated variables and is usually expressed by the correlation coefficient. When the correlation coefficient is greater than 0.95, it indicates a significant correlation between variables, and when the correlation coefficient is greater than 0.8, it indicates a high correlation between variables. Correlation analysis among the hydrochemical parameters of surface water can indicate whether the ions are homologous or not. According to the results presented in Fig. 6, the correlation coefficients of Cl^−^-Mg^2+^, Cl^−^-Na^+^, Cl^−^-K^+^, SO_4_^2−^-Ca^2+^, SO_4_^2−-^Mg^2+^, SO_4_^2−^-Na^+^, SO_4_^2−^-K^+^, Mg^2+^-Na^+^, Mg^2+^-K^+^, and Na^+^-K^+^ were all larger than 0.8, which indicates high correlation. Cl^−^ shows a high correlation with Mg^2+^, Na^+^, and K^+^, which indicates the leaching of secondary salts (Prasanna et al. 2010). The combination of these ions can easily produce insoluble salts such as CaSO_4_ and MgSO_4_, and irrigating cultivated land with such water causes insoluble salt deposits on the surface and aggravates the degree of surface salinization, thus affecting the ecological environment of the basin.

The correlation coefficients of TDS with Cl^−^, SO_4_^2−^, Mg^2+^, Na^+^, and K^+^ are all greater than 0.8, which suggests that these five ions are the main ions of surface water in the study area; among these pairings, the correlation coefficient of TDS and Mg^2+^ is the highest, indicating that Mg^2+^ plays a decisive role in the distribution of TDS in the study area, it can also be found from Table 2 that magnesium ion has the highest coefficient of variation among all ions.

The chemical composition and characteristics of water are mainly affected by two processes: the first is the dissolution and filtration of river infiltration and runoff, and the second is the evaporation and concentration of continental salinization. Water chemistry is also determined by factors such as lithology, runoff velocity, geochemical response characteristics, and human activities that cause runoff (Meybeck 1987; Zhou et al. 2014). Therefore, we need to combine the results of further statistical analysis to analyze the source of hydrochemical components.

### Source of chemical composition

Studies have shown that under natural conditions, HCO_3_^−^ and CO_3_^2−^ in water are mainly derived from the weathering and dissolution of carbonate rocks; SO_4_^2−^ and Cl^−^ are mainly derived from the weathering and dissolution of evaporated salt rock; Ca^2+^ and Mg^2+^ are mainly derived from the weathering products of carbonate, evaporite, and silicate rocks, and Na^+^ and K^+^ are mainly derived from the weathering products of silicate and evaporite rocks, another potential source of Na^+^ is the dissolution of mirabilite salt (NaSO_4_) (Sami 1992; Hussein 2004; Chen et al. 2002; Srinivasamoorthy et al. 2014; Thomas et al. 2014; Gautam et al. 2015). In arid regions, Cl^−^ and Na^+^ are mainly derived from surface-enriched salt, which increases with increasing mineralization (Zhou and Dong 2002); chloride exists in the sediments of inland lakes in arid regions, and rock salt in arid regions is an important source of Na^+^ in natural waters. Cl^−^ is a water-chemical conserved ion because in Cl-containing minerals, such as rock salt, mineral dissolution will not increase or decrease through water-rock interactions (Mazor 2003; Zhu et al. 2008; Chen et al. 2002). It is generally considered that changes in Cl^−^ concentration are only affected by evaporation-concentration or mixing with rainfall, surface water, groundwater and bedrock pore water (Hem 1985; Skrzypek et al. 2013; Dogramaci et al. 2015).

Gibbs (1970) divided the main mechanisms controlling chemical composition in water into precipitation, rock weathering, and evaporation-concentration and designed the Gibbs chart to analyze the main sources of dissolved chemical components in surface water. The ordinate of the graph is the logarithm of TDS, and the abscissa is Na^+^/(Na^+^+Ca^2+^) or Cl^−^/(Cl^−^ + HCO_3_^−^) (Marghade et al. 2012). The Gibbs diagram can intuitively indicate whether the main components of river water tend to be controlled by “Atmospheric Precipitation Dominance (APD)”, “Rock Weathering Dominance (RWD)” or “Evaporation Concentration Dominance (ECD)” and are an important means of qualitatively judging the effect of regional rocks, atmospheric precipitation, and evaporation-concentration on river hydrochemistry (Gibbs 1970). In Gibbs diagrams, a low TDS content and very high Na^+^/(Na^+^+Ca^2+^) or Cl^−^/(Cl^−^ + HCO_3_^−^) ratio (close to 1) indicate that the river is mainly recharged by atmospheric precipitation from the ocean. Such points are mainly distributed in the lower right corner of the figure, indicating that the ion composition and content are determined by the dilution effect of “pure water” on marine aerosols in the atmosphere. Intermediate values of TDS and a Na^+^/(Na^+^+Ca^2+^) or Cl^−^/(Cl^−^ + HCO_3_^−^) ratio of approximately 0.5 or less than 0.5 indicates that the ions in the river are mainly derived from rock weathering; the points of such rivers are distributed in the middle of the figure. A high value of TDS and a Na^+^/(Na^+^+Ca^2+^) or Cl^−^/(Cl^−^ + HCO_3_^−^) ratio that is also high (close to 1) indicates that the river is located in arid areas with strong evaporation; these points are distributed in the upper right corner of the figure.

**Fig. 7:**
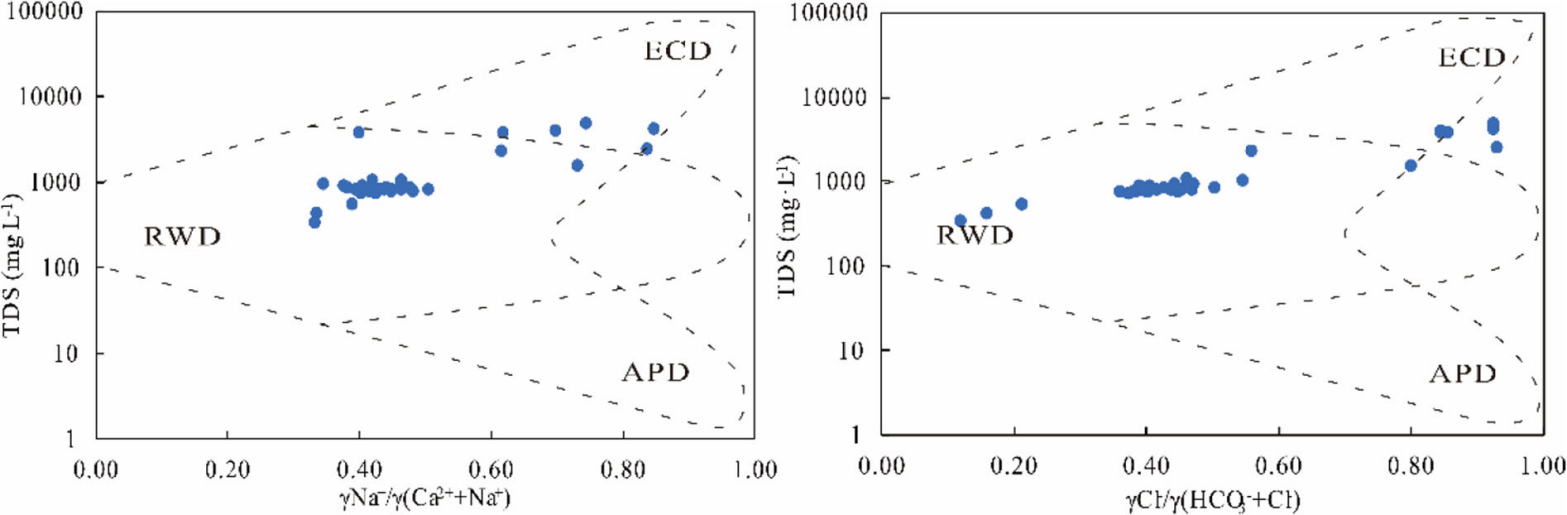
Mechanism controlling the surface water chemistry of the study area

The water chemistry data for the river water in the Kazakhstan section of the Syr Darya described in this study were plotted in a Gibbs diagram. As shown in Fig. 7, no samples were located in the lower right corner, indicating that atmospheric precipitation has no effect on the chemical concentrations in the study area; that is, the source of sea salt carried in atmospheric precipitation can be excluded. In addition, some samples are located in the rock-weathering area, and another portion of the samples is located in the evaporation-concentration area, indicating that the water chemical sources in the study area are affected by both rock weathering and evaporation-concentration, and the effect of rock weathering on the composition of hydrochemical ions is more significant.

**Fig. 8:**
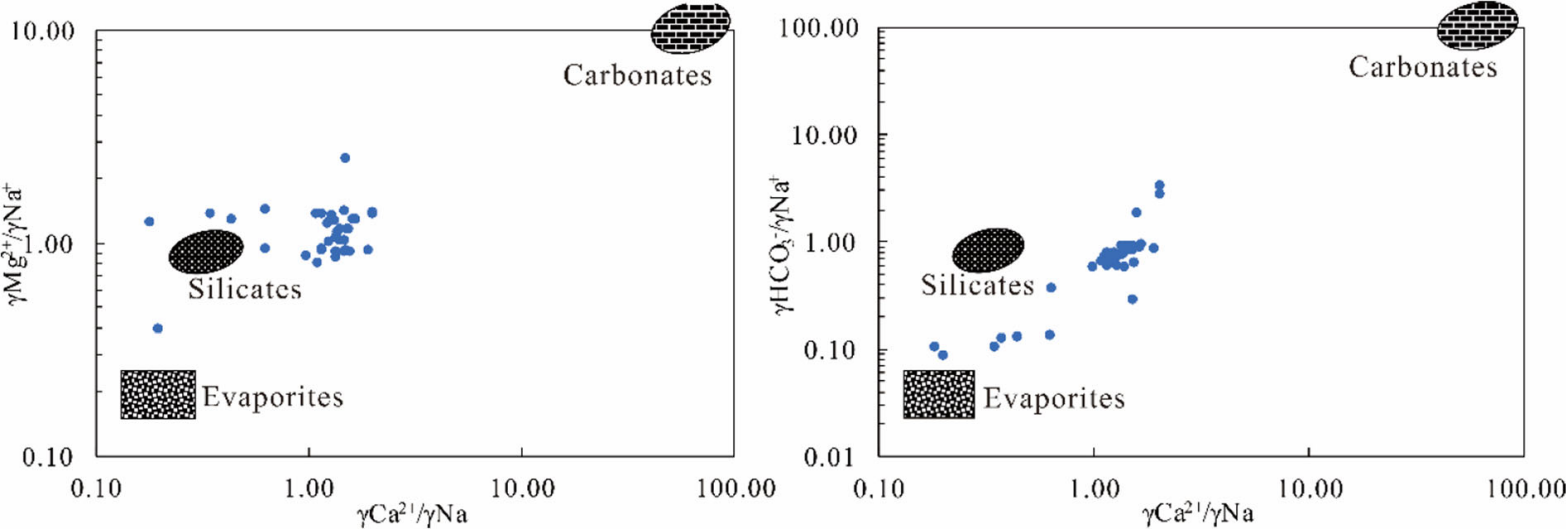
End-member diagrams of ionic ratios in surface water from the Syr Darya River in Kazakhstan

The γCa^2+^/γNa^+^, γMg^2+^/γNa^+^ and γHCO_3_^−^/γNa^+^ ratios can be further analyzed by using the end-member method to determine the type of rock weathering source associated with the surface water chemistry characteristics of the study area (Isidoro et al. 2010). The surface water data from the study area were used to create end-member diagrams of ionic ratios (Fig. 8), which show that the chemical composition of the surface water in the study area is between those of silicate rock and evaporite rock and is biased toward silicate rock, indicating that the weathering source of the chemical components of the surface water in the study area is mainly due to the weathering of silicate rock, followed by the evaporation of salt rock. This is consistent with the results in the Gibbs diagram, in which the samples are mainly in the rock-weathering control area, far from the atmospheric precipitation control area.

**Fig. 9:**
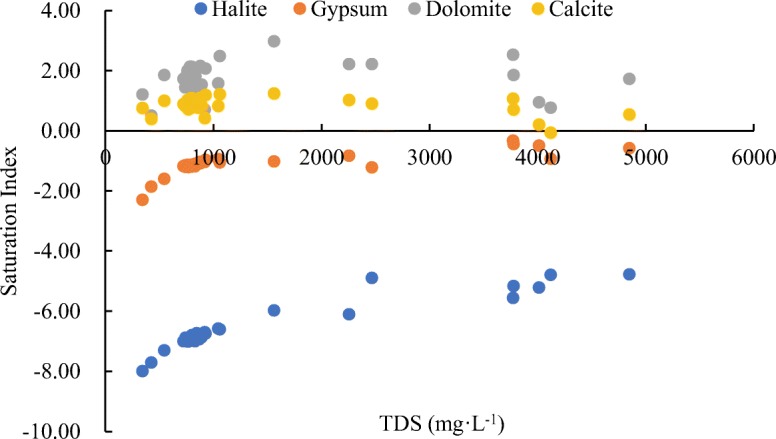
Mineral saturation index of surface water in the Syr Darya River in Kazakhstan

Figure 7 shows that the chemical composition of surface water in the study area is affected by the weathering of the rock and that the evaporation-concentration effect also has a certain influence. Evaporation-concentration tends to cause the precipitation of salts with low solubility, while salts with high solubility aggregate (Zhao 2010; Dogramaci et al. 2012). The software PHREEQC was used to calculate the saturation index (SI) of each mineral, which was used to characterize the changes in water chemical composition caused by evaporation-concentration (Version 2.12; Parkhurst and Appelo 1999) (Fig. 9). When SI > 0, it means that the mineral is supersaturated with respect to the aqueous solution; when SI = 0, it means that the mineral phase and the aqueous solution are in equilibrium; and when SI < 0, it means that the mineral is not saturated with respect to the aqueous solution and the mineral will dissolve. The formulas for the dissolution of halite (Eq. 4), gypsum (Eq. 5), dolomite (Eq. 6), and calcite (Eq. 7) are shown below:

4 $$ \mathrm{Halite}:\mathrm{NaCl}={\mathrm{Na}}^{+}+{\mathrm{Cl}}^{-} $$

5 $$ \mathrm{Gypsum}:{\mathrm{Ca}\mathrm{SO}}_4+{2\mathrm{H}}_2\mathrm{O}={\mathrm{Ca}}^{2+}+{{\mathrm{SO}}_4}^{2-}+{2\mathrm{H}}_2\mathrm{O} $$

6 $$ \mathrm{Dolomite}:\mathrm{CaMg}\ {\left({\mathrm{CO}}_3\right)}_2+{2\mathrm{CO}}_2+{2\mathrm{H}}_2\mathrm{O}={\mathrm{Ca}}^{2+}+{\mathrm{Mg}}^{2+}+{{4\mathrm{HCO}}_3}^{-} $$

7 $$ \mathrm{Calcite}:{\mathrm{Ca}\mathrm{CO}}_3+{\mathrm{CO}}_2+{\mathrm{H}}_2\mathrm{O}={\mathrm{Ca}}^{2+}+{{2\mathrm{HCO}}_3}^{-} $$

Figure 9 showed that the SIs of gypsum in all the samples in the study area were less than 0, indicating that the corresponding aqueous solutions of halite and gypsum in all the samples in the study area are unsaturated, and the SIs of calcite and dolomite in all the samples in the study area were bigger than 0, indicating that the corresponding aqueous solutions of calcite and dolomite in all the samples in the study area are oversaturated. The saturated state determines the direction of reaction that is dolomite and calcite precipitation and halite and gypsum dissolution. According to reaction equations 4 and 5, the dissolution of halite and gypsum will contribute to the production of Na^+^, Cl^−^, Ca^2+^ and SO_4_^2−^, which indicates one of the sources of these ions in the study area.

The chemical composition of water is affected by both natural and human activities. The Gibbs diagram can be used to determine the role of natural factors but cannot exclude the impact of human activities on water chemistry development; thus, the Gibbs diagram has some limitations. Pollutants emitted by human activities such as the mining, metallurgical and chemical industries and city municipal services are discharged into river water, which changes the hydrochemical composition of the water and increases the concentration of pollutants in the water, such as Cl^−^, SO_4_^2−^, and TDS (Nurlan 2017; Issanova et al. 2018; Karlykhanov and Toktaganova 2016; ZhD 2012; Nurlan 2017). In addition, humans change the hydrodynamic conditions of water when exploiting water resources and thus affect water-rock interactions or the intensity of evaporation and concentration. The Arys River is located in an area with irrigated farming and intensive animal breeding, and therefore, human activities have a substantial impact on the regional water environment.

### Irrigation suitability assessment

**Fig. 10:**
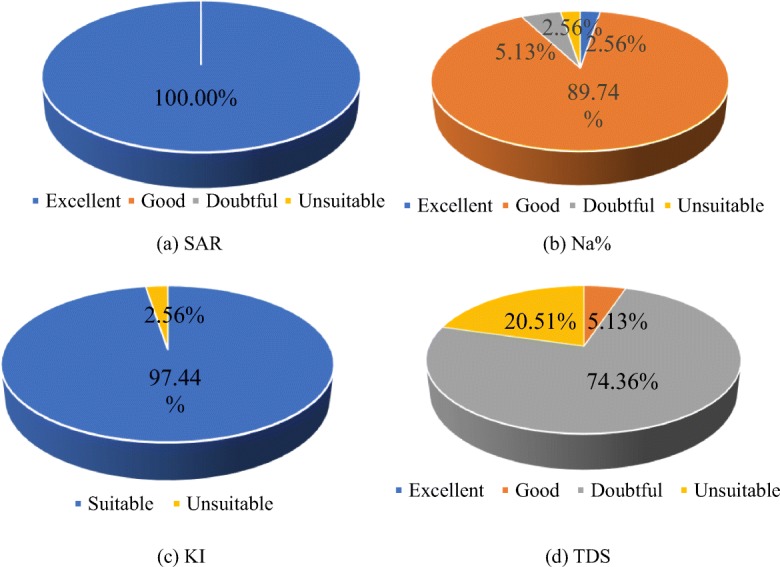
Irrigation water quality based on SAR, Na%, KI, and TDS

The high salinity content in the water is one of the main characteristics of the river water in the Syr Darya Basin, which has caused many adverse effects on the production and life of the residents and the ecological environment along the river (Létolle and Chesterikoff 1999; Bekbaev and Kazykenova 2003; Kipshakbaev et al. 2010; SIC ICWC et al. 2011; Kenjebayeva 2015). As a basic industry for national life, agriculture uses huge amounts of water resources. A large amount of salt enters farmland with irrigation water, causing crop yield reduction and soil salinization to be intensified (Bahar and Reza 2010; Ravikumar et al. 2011; Jassas and Merkel 2015). Farmland as a large proportion of landscape types, their quality changes also have a great impact on the overall landscape changes. Therefore, the suitability of irrigation based on chemical ion composition in water is discussed. Four indexes were used to analyze the irrigation suitability of water in the study area (Fig. 10). SAR represents the relative activity of Na^+^ in soil exchange reactions and is used to evaluate the degree of alkalization of irrigation water. Richards classified the irrigation suitability based on SAR as excellent (< 10), good (10–18), doubtful (19–26), and unsuitable (> 26). As shown in Fig. 10a, all samples (100%) in the study area are suitable for agricultural irrigation based on SAR values.

Na% represents the sodium hazard: the absorption of irrigation water containing high concentrations of Na^+^ into soil results in a decrease in soil permeability. The suitability of irrigation water based on Na% was classified as excellent (< 20%), good (20–40%), doubtful (40–60%), or unsuitable (60–80%). As shown in Fig. 10b, approximately 90.48% (38 samples) of water samples in the study area are good for irrigation, and only 2.38% (1 sample) of water samples are unsuitable for irrigation.

An irrigation suitability assessment of water can also be performed using KI, which is the ratio of sodium to the sum of calcium and magnesium ions. When classifying the suitability of irrigation water based on KI, a value more than 1 means that there is too much sodium in water for irrigation, and a value less than 1 means the water is suitable for irrigation. As shown in Fig. 10c, 97.62% (41 samples) of water samples in the study area were suitable for irrigation, and 2.38% (1 sample) of water samples were unsuitable for irrigation.

Based on the US Department of Agriculture laboratory classification criteria, different levels of TDS will also have an impact on the irrigation suitability of water. The suitability of irrigation water based on TDS was classified as excellent (< 150 mg L^−1^), good (150–500 mg L^−1^), doubtful (500–1500 mg L^−1^), or unsuitable (> 1500 mg L^−1^). Although the evaluation results of the above three indexes on irrigation water quality indicate that most of the sample water is suitable for irrigation, the classification results according to the mineralization content in Fig. 10d indicated that 71.43% of the water samples are in doubtful for irrigation which belongs to the range of boundary values, 23.81% are unsuitable for irrigation, and only 4.76% are good for irrigation. This indicates that the influence of TDS concentration on irrigation water quality in the study area cannot be ignored.

## Conclusion

The surface water of the Syr Darya River within Kazakhstan is weakly alkaline, and the TDS content in the water shows that 25.64% of the water is salt water.

The main anion of surface water in the study area is SO_4_^2−^, and the main cations are Na^+^, Ca^2+^ and Mg^2+^. The ions Cl^−^, SO_4_^2−^, Mg^2+^, Na^+^, and K^+^ have a great influence on the salinity concentration. The main water chemistry type is Ca-Mg-SO_4-_Cl, which accounted for 89.74% of water samples.

From the perspective of natural factors, the hydrochemistry in the study area is derived from the dual effects of rock weathering and evaporation and concentration. The analysis results indicated that it mainly the dissolution of gypsum and halite, and weathering of silicate.

From the perspective of anthropogenic factors, the hydrochemistry in the study area is determined by industrial and agricultural production near the river and the inflow of urban domestic sewage. The large-scale use of return water containing multiple pollutants is the main reason for the high chemical content of water in the study area.

The irrigation suitability evaluation of surface water in the study area based on SAR, Na% and KI showed that the majority of the water is suitable for irrigation and that only a small part of the water is not suitable for irrigation, and from the TDS content analysis, 71.43% of the samples are in a critical state, indicating that the influence of TDS concentration on irrigation suitability cannot be ignored.

## Electronic supplementary material

ESM 1 (DOCX 14 kb)
