# Supplementary material for: Hydrochemical characteristics and irrigation suitability of surface water in the Syr Darya River, Kazakhstan
Source: Environ Monit Assess. 2019 Aug 16;191(9):572. doi: 10.1007/s10661-019-7713-8 (PMC6697754; doi:10.1007/s10661-019-7713-8)
Supplement: Supplementary file 1 — (DOCX 14 kb) [file 10661_2019_7713_MOESM1_ESM.docx]

| Sample No. | Sample position | Longitude | Latitude |
| --- | --- | --- | --- |
| 1 | Shardara reservoir | 67.9103 | 41.2521 |
| 2 | Main channel | 67.9515 | 41.2652 |
| 3 | Main channel | 68.2491 | 42.2213 |
| 4 | Small tributary | 68.3014 | 42.2223 |
| 5 | A catchment for water from sample No.4 | 68.4596 | 42.2361 |
| 6 | Arys river | 68.9507 | 42.4644 |
| 7 | Bugunskoye water reservoir | 69.1477 | 42.7518 |
| 8 | Shoshkakol lake | 68.4145 | 43.1167 |
| 9 | Main channel | 68.2168 | 42.6140 |
| 10 | A bifurcated channel through the residential area of Shaulder | 68.3669 | 42.7570 |
| 11 | Main channel | 67.8516 | 43.1590 |
| 12 | Main channel | 67.7790 | 43.2288 |
| 13 | Main channel | 67.5068 | 43.5454 |
| 14 | Koylekata Lake,receiving irrigation water | 67.3327 | 43.6666 |
| 15 | Main channel | 67.1996 | 43.8647 |
| 16 | Main channel | 66.7101 | 43.9505 |
| 17 | Main channel | 66.3712 | 44.2774 |
| 18 | Small tributary | 66.2677 | 44.2926 |
| 19 | Small tributary | 66.1589 | 44.3628 |
| 20 | Main channel | 65.6304 | 44.7237 |
| 21 | Main channel through the residential area | 65.5105 | 44.7827 |
| 22 | Small tributary beyond farmland | 65.4285 | 44.8161 |
| 23 | Small tributary beyond farmland | 65.3179 | 44.8863 |
| 24 | Main channel | 65.2701 | 44.9844 |
| 25 | A lake | 65.3670 | 45.0192 |
| 26 | Small tributary | 64.9905 | 44.8992 |
| 27 | Small tributary | 64.9891 | 45.0175 |
| 28 | Small tributary | 64.6524 | 45.0483 |
| 29 | Main channel | 64.4726 | 45.1118 |
| 30 | Small tributary | 64.2307 | 45.4537 |
| 31 | Main channel | 64.0653 | 45.4685 |
| 32 | Qamystybas lake | 61.9304 | 46.2118 |
| 33 | Qamystybas lake | 61.6611 | 46.1468 |
| 34 | Small lake | 61.2651 | 46.1477 |
| 35 | Small lake | 61.0859 | 46.0754 |
| 36 | Main channel | 60.8667 | 46.1019 |
| 37 | Main channel | 61.0528 | 46.0244 |
| 38 | Kokaral dam | 60.7679 | 46.1022 |
| 39 | North Aral Sea | 60.8473 | 46.1521 |
